# A Laser-Induced Graphene-Titanium(IV) Oxide Composite for Adsorption Enhanced Photodegradation of Methyl Orange

**DOI:** 10.3390/nano13050947

**Published:** 2023-03-05

**Authors:** Brhane A. Tesfahunegn, Maurício Nunes Kleinberg, Camilah D. Powell, Christopher J. Arnusch

**Affiliations:** Department of Desalination and Water Treatment, Zuckerberg Institute for Water Research, The Jacob Blaustein Institutes for Desert Research, Ben-Gurion University of the Negev, Sede-Boqer Campus, Midreshet Ben-Gurion 8499000, Israel

**Keywords:** laser-induced graphene, titanium(IV) oxide, nanocomposite, adsorbate, photocatalyst, emerging contaminants

## Abstract

Numerous treatment methods such as biological digestion, chemical oxidation, and coagulation have been used to treat organic micropollutants. However, such wastewater treatment methods can be either inefficient, expensive, or environmentally unsound. Here, we embedded TiO_2_ nanoparticles in laser-induced graphene (LIG) and obtained a highly efficient photocatalyst composite with pollutant adsorption properties. TiO_2_ was added to LIG and lased to form a mixture of rutile and anatase TiO_2_ with a decreased band gap (2.90 ± 0.06 eV). The LIG/TiO_2_ composite adsorption and photodegradation properties were tested in solutions of a model pollutant, methyl orange (MO), and compared to the individual and mixed components. The adsorption capacity of the LIG/TiO_2_ composite was 92 mg/g using 80 mg/L MO, and together the adsorption and photocatalytic degradation resulted in 92.8% MO removal in 10 min. Adsorption enhanced photodegradation, and a synergy factor of 2.57 was seen. Understanding how LIG can modify metal oxide catalysts and how adsorption can enhance photocatalysis might lead to more effective pollutant removal and offer alternative treatment methods for polluted water.

## 1. Introduction

Emerging contaminants (ECs) have recently been found in aquatic habitats all over the world. Their detrimental effects on health are not fully understood [1]. Over the past ten years, studies on contaminants have grown to better understand these effects. Several industrial organic compounds, personal care items, pesticides, endocrine-disrupting complex chemicals, and active medicinal chemicals are examples of these pollutants [2,3]. ECs can enter the environment and have known or suspected negative consequences for the ecosystem and/or human health. Numerous wastewater treatment methods have been developed, including coagulation, biological digestion, and chemical oxidation. These techniques can suffer from low efficiencies, are complex, or are not economically feasible [4]. Adsorption technology has many benefits, including affordability, effectiveness, and ease of use [5,6]. Minerals [7], silica [8], carbon materials [9], metal-organic frameworks [10], and granular activated carbon [11] are typical adsorbent materials. However, the pollutants are not converted into non-polluting chemicals during the adsorption process; rather, they are simply transported from the aqueous phase to the solid phase. In this situation, wastewater treatment can result in secondary contamination [5]. On the other hand, heterogenous photocatalysis can potentially remove organic contaminants from the environment with high efficiency [12]. The process in its most efficient form can detoxify organic pollutants by transformation to water and carbon dioxide, and thus no harmful byproducts or secondary pollution streams are formed [5]. A combinational technique based on both the adsorption and photocatalysis methods has been devised to address the constraints of adsorption and photocatalysis [13,14]. Adsorption considerably improves the interface between a pollutant and a photocatalyst, resulting in enhanced photocatalytic activity, and the adsorbed organic contaminations can be successfully destroyed and mineralized by photocatalysis [5].

A broad spectrum of metal oxide semiconductor materials can be used as photocatalysts, including Ce-doped ZnO [15], ZnS, ZnO, TiO_2_, CdS, WO_3_, and AgPO_4_ [16,17,18]. It has been shown that supporting ZnO/ZnS on the surface of biochar led to the expansion of the active region. Consequently, catalytic activity was increased [19]. It is essential to maintain a reasonable balance between adsorptive and catalytic sites to maximize the synergistic benefits of both processes. A typical heterogeneous photooxidation reaction generates electrons in the conduction band and holes in the valence band, provided that the photons have equal or higher energy than the band gap energy of the photocatalyst. The electrons in the conduction band yield superoxide anions (**•**O_2_^−^) after reacting with oxygen (O_2_), and hydroxyl radical (**•**OH) upon contact with water (H_2_O), via the holes in the valence band [20]. In comparison to traditional methods, the advantages of heterogeneous photocatalysis include its low operational costs, effectiveness, and fast reaction times. N-type metal oxides are sometimes preferred as catalysts over noble metal oxides for many reasons. Ti, Zn, and Sn are common and abundant elements in intrinsic n-type metal oxides, while Pt, Pd, and Rh are noble metals that are comparatively more rare and expensive. Additionally, n-type metal oxides are tunable; by altering their composition, morphology, and surface area, intrinsic n-type metal oxides can have their properties tailored, which can be crucial for improving the efficiency of catalysts [21]. In addition to that, intrinsic n-type metal oxides are more stable than catalysts based on noble metals. They are less likely to leak or lose their effectiveness over time, which might be crucial in challenging environments or for prolonged use. Furthermore, in comparison to noble metals-based catalysts, intrinsic n-type metal oxides frequently exhibit superior selectivity for processes. They can therefore be utilized to generate more targeted products with fewer undesirable byproducts [22]. In the area of photocatalysis for pollutant removal, titanium oxide (TiO_2_) is widely studied because of its low toxicity, good chemical stability, high photo activity, and low material cost [16,23,24,25].

A notably intrinsic n-type metal oxide semiconductor (anatase TiO_2_) has a large band gap of 3.2 eV; this limits the use of a wide spectrum of light for photocatalysis. Additionally, TiO_2_ micro- or nanomaterial based catalysts are also difficult to recover [16], and solutions include TiO_2_ modification using various techniques such as semiconductor loading, ion doping, metal deposition, and semiconductor recombination [26,27,28,29]. Moreover, the photocatalytic performance of TiO_2_ is decreased because of the exciton decay and the photo generated electron and hole parasitic recombination [30]. The carbon atomic structure and its nanostructure enhances the photocatalytic activity of TiO_2_ through one or all of the three main mechanisms: band gap tuning, minimization of electron/hole recombination, and the quality and quantity of suitable active sites [31]. Carbon-based materials such as graphene, carbon nanotubes (CNT), graphene oxide, and reduced graphene oxide have been combined with different metal oxide semiconductors for enhanced photocatalytic effects [30,32,33].

Activated carbon has been used as a support for TiO_2_ [26]; nevertheless. the presence of a huge number of microporous structures could possibly hinder the transport of micropollutants towards the catalyst active site, and the pore might be masked by TiO_2_ [24]. It has been reported that carbon black could be a better supporting material for TiO_2_ nanoparticles [24]. The authors reasoned that carbon black has a bigger pore structure than activated carbon and has a higher electrical conductivity. Carbon quantum dots have been used on their own as a catalyst for photopolymerization [34]. In general, however, graphene is a two-dimensional allotrope of carbon and has received extensive research interest worldwide due to its excellent electronic properties, thermal conductivity, mechanical strength, high optical transmittance, and good interfacial interactions with various adsorbents [35,36,37], and it can be combined in various ways with metal nanoparticles [38].

The work of Ahmad et al. in 2013 showed that a composite of ZnO and graphene has outstanding photocatalytic performance due to graphene′s good charge separation capability [30,39]. Additionally, it is possible to improve the catalytic performance of graphene-based semiconductor photocatalysts by modifying the structure or chemical composition of graphene to make it more porous and avoid potential diffusion limited transport schemes of the pollutant to the active sites within the material. Thus, among other possible electrically conductive and porous carbon nanomaterials, laser-induced graphene (LIG) might be an ideal support material. Its ease of fabrication combined with its low cost has resulted in its incorporation into numerous electrical applications, such as energy storage, adsorption studies, biosensors, and composites [40,41,42].

**Figure 1:**
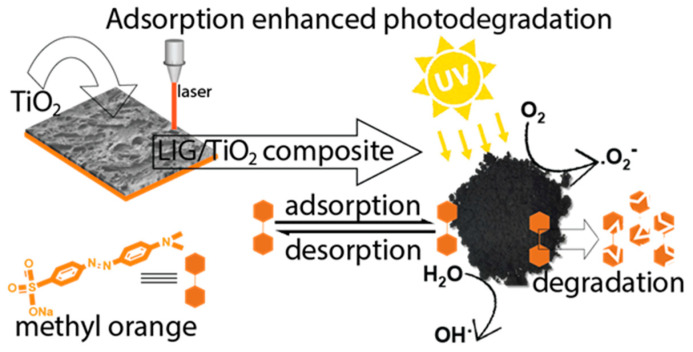
Schematic illustration of the fabrication of the LIG/TiO_2_, adsorption and the photodegradation process of methyl orange.

In this work, we developed an LIG/TiO_2_ composite using a double lasing method starting with a commercial polyimide substrate (Figure 1). This method was quick, cheap, environmentally friendly, and did not require the extensive use of chemicals. The LIG/TiO_2_ composite was tested for the adsorption and photocatalytic degradation of an organic dye: methyl orange, a model pollutant. The chemical composition, morphology, surface charge, surface area, and band gap were evaluated. Lastly, the decolorization, mineralization, adsorption thermodynamics, degradation kinetics, and catalyst reusability were carefully investigated.

## 2. Experimental Section

### 2.1. Materials

A ~120 µm thick polyimide substrate was purchased from Runsea industry (HK), Guangdong, China. Anatase titanium (IV) oxide with particle size < 25 nm, methyl orange (MO) with 85.0% dye content, and hydrochloric acid (37.0%) was purchased from Sigma Aldrich, MO, USA. Technical grade, 99.9% ethanol was purchased from Romical, Beersheba, Israel. Sodium hydroxide (97.0%) was purchased from Biolabs, Jerusalem, Israel. Deionized water (DI) was obtained from a Milli-Q ultrapure water purification system (Millipore, Billerica, MA, USA).

### 2.2. Preparation of LIG/TiO_2_ Composite

A 25 cm × 25 cm polyimide (PI) substrate was attached to the processing area of a 10.6 μm CO_2_ laser cutting system (Figure S1a,c). An air nozzle directed toward the lens was activated to keep the lens free of material dusts. The substrate was then irradiated at specific laser operating parameters under ambient conditions. To fabricate LIG or LIG/TiO_2_, the laser power and scanning speed were set to 10.0% and 30.0%, respectively, with the laser pulses per inch (PPI) set to 1000. Other operational parameters, such as image density and vector, were set to 6 and Q, respectively (Figure S1b). First, LIG on polyimide was obtained after first lasing. Next, TiO_2_ (100 mg) that was mixed in ethanol (15 mL) was drop casted on the laser induced graphene surface, and after evaporation of the ethanol the average surface density of TiO_2_ was equal to 0.16 mg/cm^2^. Subsequently, the TiO_2_ coated LIG was irradiated with the same laser settings which embedded the nanoparticles within its multilayer matrix. Then, the LIG/TiO_2_ composite powder was obtained by carefully scraping the carbonized part of the PI film. An LIG/TiO_2_ suspension was then prepared by mixing 7.5 mg of the composite powder with 15 mL DI and the solution was sonicated for ~30 min in a D-74224, Elma Singen bath sonicator.

### 2.3. Characterization

Scanning electron microscopy (SEM), equipped with FEI JEOL IT 200, was used to study the morphology of the composite and perform energy dispersive X-ray analysis (EDS) characterization. Samples were mounted and sputter coated with ultra-thin conducting film Au-Pd before visualization with SEM. A transmission electron microscope (TEM), Tecnai T12 200 kV TEM with 120 kV, was used during surface imaging. Scraped LIG/TiO_2_ was suspended in DI water and sonicated for 30 min. X-ray diffraction (XRD) patterns were recorded with a model-Rigaku D/max ultima II, equipped with an X-ray source with 0.1541 nm wavelength, using Cu-Kα radiation. XRD measurements were conducted at constant current of 100 mA and voltage of 45 kV.

X-ray Photoelectron Spectroscopy (XPS) revealed the chemical composition of the LIG/TiO_2_ composite. For XPS measurements, an ESCALAB 250 (Thermo Fisher Scientific, Waltham, MA, USA) with an ultrahigh vacuum at 1 nano bar installed with an Al kα X-ray source (beam size of 0.5 mm) was used. Signals from the Ti_2p_, C_1s_ and O_1s_ were detected by fixing different separated elements to the experimental data. Samples were dried for 12 h before XPS characterization. Raman spectroscopy with a Horiba LabRam HR evolution micro-Raman system, equipped with a Synapse Open Electrode with charge coupled detector air-cooled to −60 °C, was used. A general laser with wavelength of 532 nm and power 0.5 mW was used as a light source for the spectrometer. LIG and LIG/TiO_2_ samples were exposed for Raman scanning for 30 s. Results were recorded using 100 μm confocal microscope hole and 600 grooves/mm grating set.

The zeta potential of anatase TiO_2_, LIG, and LIG/TiO_2_ was analyzed using a Nano ZS equipped with He-Ne Laser 633 nm, maximum power 5 mW, and instrument critical operational conductivity of the suspension 200 mS/cm. Surface charge was measured by varying the pH of suspension from 1.5 to 12. UV-Vis absorption measurement was used to estimate the band gap of the composite. For that, LIG/TiO_2_ powder, which was scraped after the laser treatment, was dispersed in DI water. This solution of LIG/TiO_2_ was added to a quartz cuvette with 1 cm path length, and the absorbance spectrum from 200 nm to 800 nm was measured. Surface areas of LIG, TiO_2_ and LIG/TiO_2_ were measured using NOVAtouch Brunauer–Emmett–Teller (BET) with the following specifications: surface area range (1 m^2^/g—unknown upper limit), pore size (2–500 nm) and temperature range (ambient—450 °C, 1 °C intervals). It was equipped with Nitrogen gas (N_2_) as adsorbate with a relative pressure of 6 × 10^−8^ mm Hg.

### 2.4. Adsorbate

Methyl orange (MO), with a maximum absorption at a wavelength of 464 nm in the UV-Vis spectrophotometer, was used as a model pollutant. A stock solution with 1 g L^−1^ MO was prepared by dissolving 1 g of MO powder in 1 L of DI. Solutions with different concentrations were prepared by diluting the stock solution with DI.

### 2.5. Effect of Initial Concentration

The effect of initial MO concentration on LIG/TiO_2_ sorption capacity and removal efficiency was evaluated. Solutions with initial MO concentrations of 5 mg/L 20 mg/L, 50 mg/L, and 80 mg/L were prepared. For all experiments, a 7.5 mg dosage of LIG/TiO_2_ was added to 15 mL of a MO solution in the absence of light and under ambient conditions. To reach adsorption–desorption equilibrium, samples were thoroughly mixed with a Roto-Therm plus (i.e., 30 rpm for 3 h). Samples were then filtered through a 20 µm filter before UV-visible spectrophotometer analysis. MO concentrations were measured before and after adsorption at 464 nm. This exact same experimental procedure was repeated using LIG/TiO_2_. Adsorption capacity was calculated with Equation (1), while sorption percentage (%) was calculated with Equation (2). *C_o_* is the initial concentration of MO (mg/L), *C_e_* is the equilibrium concentration of the MO (mg/L), *W* is the mass of absorbent (g), *q_e_* is the adsorbed amount per unit gram of absorbent (mg/g), and *V* is the volume of the solution in (L).

(1) $$q_{e}=\frac{\left(C_{o}-C_{e}\right)V}{W}$$

(2) $$\mathrm{Sorption}\;(\%)=\frac{\left(C_{o}-C_{e}\right)*100}{C_{o}}$$

### 2.6. Effect of Solution pH

The effect of solution pH was investigated by varying the pH of the MO solution from 2 to 11. Solutions of 0.1 M NaOH or 0.1 M HCl were used to adjust pH. To study the effect of pH on rate of sorption, the dose of LIG/TiO_2_ was fixed at 0.5 mg/mL while the initial concentration of MO was varied from 20 mg/L to 100 mg/L. Said concentrations were chosen to investigate the effect of pH because complete removal was observed at concentrations lower than 20 mg/L and low pH.

### 2.7. Effect of LIG/TiO_2_ Dose

The LIG/TiO_2_ dose was varied (i.e., 60 mg/L, 125 mg/L, 250 mg/L, 500 mg/L, and 1000 mg/L) to study the effect of LIG/TiO_2_ dose on MO adsorption. The pH of each solution was adjusted to 7.0. For each experiment, temperatures were set to 298 K and all solutions were kept in the dark.

### 2.8. Adsorption Isotherms

To predict the adsorption capacity of the adsorbent and to understand/analyze the possible mechanisms of the adsorption process, various adsorption isotherms were studied. Langmuir, Freundlich and Temkin adsorption isotherms were chosen for the analysis, given that they are the most common adsorption isotherms in liquid phase solution and carbon materials as adsorbent. For the Langmuir isotherm, the maximum adsorption capacity is achieved due to a monolayer of adsorbate (in this case MO) being adsorbed onto the homogeneous sites of adsorption on the surface of a selected adsorbent. On the other hand, Freundlich isotherms assume the presence of heterogeneous sites of adsorption. Moreover, the Freundlich isotherm assumes that physical adsorption happens in such a way that pollutant molecules form a multilayer structure on the surface of the adsorbent. Lastly, the Temkin isotherm model assumes that the heat of adsorption for each pollutant molecule decreases as the surface coverage of the adsorbent increases. Additionally, it considers the presence of an even binding energy distribution on the adsorbent′s surface. The linearized equations and plotting for each isotherm model are given in Table 1.

### 2.9. Adsorption Thermodynamics Study

The role of overall entropy (ΔS), overall enthalpy (ΔH), Gibbs free energy (ΔG), and temperature on the adsorption process of MO by LIG/TiO_2_ were investigated. For these studies, the following initial concentrations of MO were chosen: 5 mg/L, 20 mg/L, 50 mg/L, and 80 mg/L. Subsequently, 7.5 mg of LIG/TiO_2_ was added to 15 mL of each MO solution. Solutions were thoroughly mixed with a Roto-Therm plus at 30 rpm and incubated at three desired temperatures (298 K, 318 K, 333 K). Each experiment was carried out at atmospheric pressures, in dark conditions, and conducted in triplicates.

### 2.10. Photocatalytic Degradation Study

The photocatalytic tests were performed using an Omni Cure LX500 instrument with a wavelength of 365 nm. Temperature and pressure were maintained at 298 K and 1 atm, respectively. In this photodegradation experiment, the MO concentration and irradiation time was set to 20 mg/L and 10 min, respectively. Likewise, for the adsorption thermodynamics and isotherm studies, 0.5 mg LIG/TiO_2_ per 1 mL of MO solution was applied for the photocatalytic test. To ignore the effects of adsorption, the samples were allowed to reach adsorption–desorption equilibrium before being exposed to any irradiation. Separate samples were prepared for each exposure time. Even though TiO_2_ and LIG/TiO_2_ have a low absorbance at 464 nm, their presence can affect the accuracy of our measurements by blocking or reflecting light. Therefore, to prevent this, 10 mL solution from each sample was filtered using a 20 µm filter and subsequently taken for centrifugation. The resultant supernatant was then used to study the dye (model pollutant) degradation kinetics.

## 3. Results and Discussion

**Figure 2:**
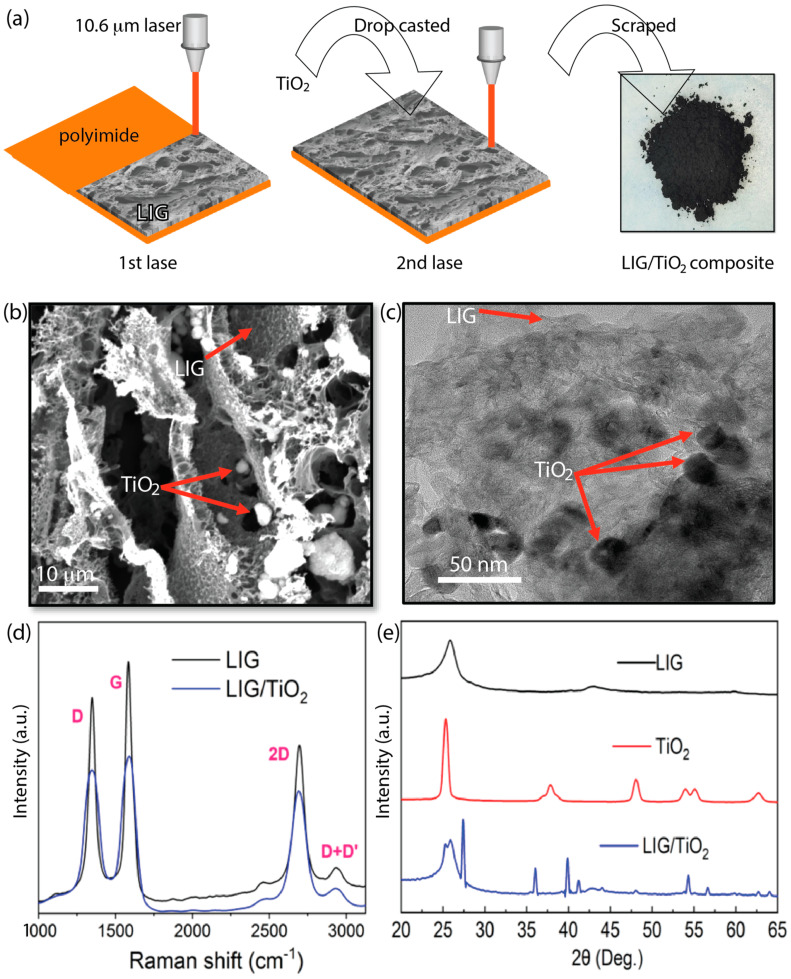
(**a**) Step by step fabrication of LIG/TiO_2_ composite; (**b**) SEM image showing surface morphology of LIG/TiO_2_; (**c**) TEM image of LIG/TiO_2_ composite; (**d**) Raman spectrum of both LIG and LIG/TiO_2_ showing D, G, 2D and D+D’ peaks; (**e**) X-ray diffraction (XRD) patterns of LIG, TiO_2_, and LIG/TiO_2_.

Electrically conductive LIG was fabricated by direct laser treatment of polyimide under ambient conditions (Figure S2). As shown in Figure 2a, the same lasing settings are used to embed titanium oxide nanoparticles between graphene layers. The scraped powder was characterized and tested for both adsorption and photocatalytic degradation of MO. Elemental mapping and surface morphology examination were performed using SEM. Aggregates of TiO_2_ nano particles with different sizes were distributed between the layers of LIG (Figure 2b). To confirm the presence and the distribution of TiO_2_ nanoparticles on the surface and between layers of the LIG, EDS was carried out for qualitative and quantitative analysis. According to the manufacturer, the TiO_2_ nanoparticles were 25 nm. However, once the solution was drop casted on the LIG and released to form the composite, the SEM images revealed the formation of bigger aggregates.

High-resolution TEM images confirm the presence of embedded TiO_2_ nano particles between the layers of the LIG, indicating close physical contact (Figure 2c; Figure S3a,b). Previous studies have also shown that metal nanoparticles can form and embed in LIG [43]. The graphene layers can be easily differentiated from the TiO_2_ nano particles that had an average size of approximately 25 nm. The Raman spectra showed a G peak at 1584 cm^−1^, D peak at 1349 cm^−1^, 2D peak at 2694 cm^−1^ and D+D’ peak at 2940 cm^−1^ (Figure 2d). The I_D_/I_G_ ratio of pristine LIG was 0.85, compared to 0.93 for LIG/TiO_2_, and indicated that the structural defects in the composite were slightly increased. The ratio of the D and G band is highly affected by electrons and hole doping [44]. The I_2D_/I_G_ (0.78) of the LIG alone revealed the presence of multilayered graphene.

XRD patterns of the LIG, TiO_2_, and LIG/TiO_2_ (Figure 2e) were measured for a deeper understanding of the phases and grain sizes and to determine the ratio of different crystal polyforms. Titanium(IV) oxide alone has three main crystal polyforms, including anatase, rutile and brookite [45,46]. The XRD profile of LIG showed a peak at 2θ = 25.87°, corresponding to the 002 plane, and a weak peak located at 2θ = 42.89°, representing the 100 plane corresponding to a high level of graphitization and in-plane structure LIG, respectively [47]. The well-defined sharp XRD profile of TiO_2_ alone is characteristic of a material with good crystallinity. The presence of the anatase phase within the TiO_2_ was indicated by the Bragg diffraction peaks at 2θ = 25.25° and 2θ = 48.00°, both represented by Miller indices of 101. On the contrary, the occurrence of rutile phase TiO_2_ is indicated by Bragg diffraction peaks at 2θ = 27.41° and 2θ = 54.48°, represented by Miller indices of 110 [48,49].

Since no peak was identified at those specific Bragg diffraction angles, we concluded that there was no measurable rutile in the anatase TiO_2_ powder (Figure 2e, red line). However, after lasing, both the anatase and the rutile forms were observed in the composite (Figure 2e, blue line). The ratio of anatase to rutile, or vice versa, can be estimated using the Spurr–Meyers Equations (3) and (4) [50], where *I_R_* is the intensity of the rutile peak at 2θ = 27.41°, and *I_A_* designates the anatase phase intensity at 2θ = 25.25°.

(3) $$X_{A}\left(\%\right)=\frac{100}{1+1.265\frac{I_{R}}{I_{A}}}$$

(4) $$X_{R}\left(\%\right)=\frac{100}{1+0.8\frac{I_{A}}{I_{R}}}$$

Both *X_A_* and *X_R_* represent the weight percentage of anatase and rutile, respectively. In accordance with Spurr–Meyers equations, *X_R_* (%) in our samples were 0%, 0%, and 64.0% in LIG, TiO_2_, and LIG/TiO_2_, respectively. Laser treatment has shown the conversion of the anatase phase to the rutile phase in TiO_2_; for example, the laser-induced anatase to rutile transition [51]. This is further confirmed by the XRD spectra (with identified phase) in the supporting information (Figure S4).

**Figure 3:**
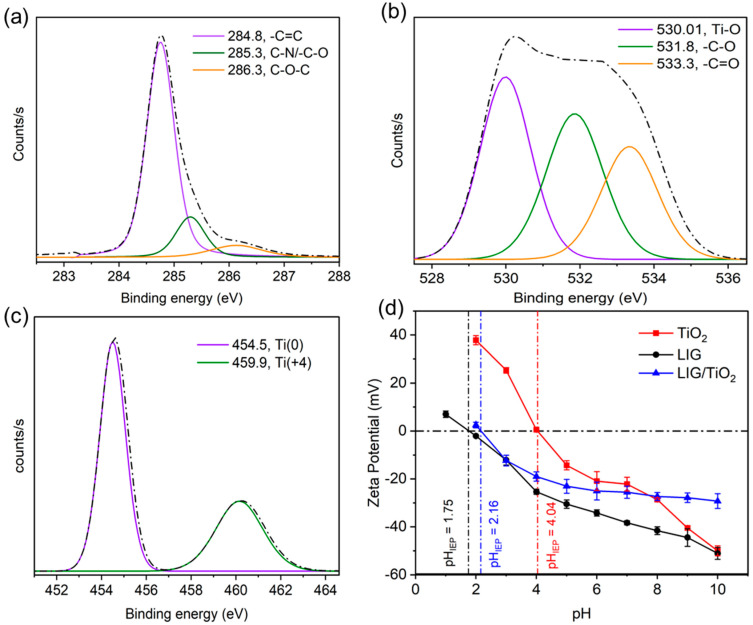
Deconvolution of (**a**) C1s, (**b**) O1s, (**c**) Ti2p; (**d**) zeta potential measurement of LIG, TiO_2_, and LIG/TiO_2_.

XPS analysis provided insight into the surface atomic structure of the newly synthesized LIG/TiO_2_ adsorbent and photocatalyst (Figure 3a–c). High resolution XPS scans of the C1s, O1s and Ti2p regions were investigated, and were fitted with a symmetrical Gaussian/Lorentzian function [52]. For all XPS spectra shown in Figure 2a–c, the background corrections were undertaken using Shirley, Model, ARbitrary, and Tougaard (SMART) because of the complex shape, and were not well approximated using a straight line. SMART background correction is a method developed to address these issues and involves fitting a mathematical function to the background signal. The Shirley model is a widely used function that describes the smooth, low-intensity background signal in XPS spectra, while the Model, ARbitrary, and Tougaard methods are alternative functions that can be used to fit more complex backgrounds. All corresponding binding energies were calculated by calibrating and fixing the C1s to binding energy of 284.8 eV. The C1s was deconvoluted to three peaks, C=C at the binding energy of 284.8 eV, C-N/C-O at 285.3 eV, and C-O-C at 286.3 eV (Figure 3a) [53,54,55,56]. O1s was deconvoluted to show three strong peaks, located at 530.01 eV, 531.8 eV, and 533.3 eV corresponding to Ti-O, C-O, and C=O, respectively (Figure 3b). The Ti2p peak was deconvoluted into two peaks, one located at 454.5 eV and the other at 459.9 eV, attributed to Ti^0^ and Ti^4+^ (the main valence state in TiO_2_), respectively, which agree with previously reported works [53,57,58].

The zeta potential for LIG, TiO_2_ and the LIG/TiO_2_ composite was measured at variable pH (Figure 3d). The isoelectronic point (IEP) was found to be 1.75, 2.16, and 4.04 for LIG, LIG/TiO_2_, and TiO_2_ nanoparticles, respectively. Thus, the LIG/TiO_2_ composite was slightly less negative than LIG alone, which might cause minor differences in adsorption capacity at variable pH. The pH causes charge differences in functional groups within the adsorbent, which can change the adsorbent’s surface charge and affect electrostatic interactions with a charged pollutant. As the pH increases from two to eleven, the surface charge of the LIG alone and the LIG/TiO_2_ composite becomes more negative. This can eventually lead to increased electrostatic repulsion with negatively charged pollutant molecules such as anionic dyes. The BET surface area of LIG/TiO_2_ was estimated to be 113.6 m^2^/g. Besides the surface area of the adsorbent, electrostatic interactions, hydrophobicity and π-π interactions play important role during adsorption process [47].

### 3.1. Adsorption Isotherms and Thermodynamic Study

**Figure 4:**
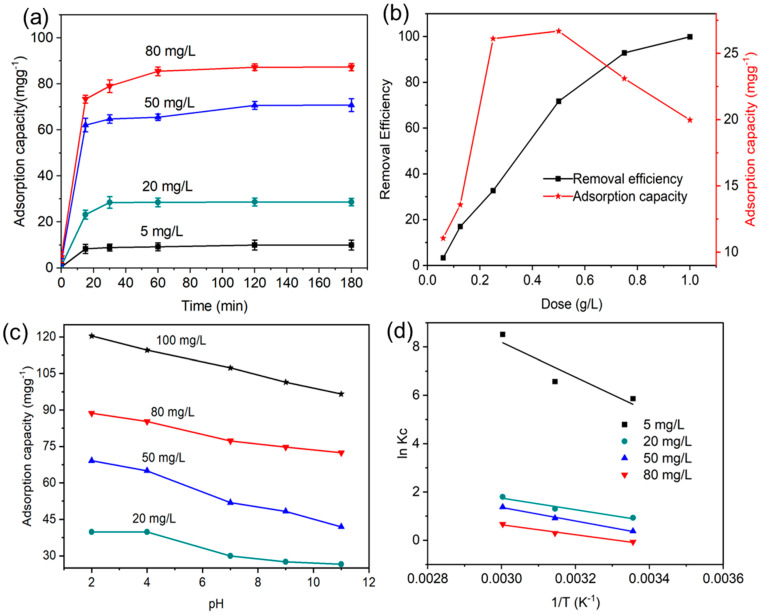
(**a**) Adsorption capacity versus contact time for various initial MO concentrations at T = 298 K; (**b**) effect of increasing dose of adsorbent on removal efficiency and adsorption capacity; (**c**) Effect of pH on the adsorption capacity of LIG/TiO_2_; (**d**) plots of LnK_c_ versus 1/T for the adsorption of MO on LIG/TiO_2_ at different dye concentrations.

To understand whether the photocatalytic properties of the LIG/TiO_2_ composite can be enhanced through an adsorption effect, the adsorption isotherm and thermodynamic studies were performed. In addition, adsorption kinetic models (pseudo first order, pseudo second order, and Elovich) were assessed (Figure S5a–c). The adsorbate (MO) concentration and the LIG/TiO_2_ composite dose were varied, and the adsorption capacity was calculated. The adsorption capacity of the composite was studied at different concentrations of methyl orange (5 mg/L, 20 mg/L, 80 mg/L) at 298 K and neutral pH. As shown in Figure 4a, increasing MO concentration resulted in an increase in adsorption capacity. The adsorption and desorption equilibriums were sufficiently reached after 3 h in dark conditions. For example, at 5 mg/L MO, the adsorption capacity after 15 min and 180 min was found to be 8.3 mg g^−1^ and 10 mg g^−1^_,_ respectively. The adsorption increased rapidly from 0 to 15 min, but then only minor increases were seen until t = 3 h. In comparison, the adsorption capacity for 20 mg/L MO was found to be 28.7 mg.g^−1^ after 3 h. The higher MO concentration leads to higher advective mass transfer in the system and increases the solute concentration gradient between the solution and the solid surface in the aqueous system interface. The synthesized composite was able to remove 71.7% of the original MO concentration by adsorption only.

Next, we investigated the effect of different composite doses on the adsorption capacity and removal efficiency because an optimized adsorption process might play an important role in the photocatalysis process. The dosage of the composite LIG/TiO_2_ catalyst was increased from 0.06 g/L to 1 g/L. The slope of the adsorption capacity curve (Figure 4b, black line) increased from 0.06 g/L to 0.25 g/L but begins to decrease from 0.25 g/L to 1 g/L. The removal efficiency increased as we increased the dosage because of the increased number of active sites in the catalyst surface [59]. Nevertheless, we observed the adsorption capacity increase till 0.5 g/L and then decrease when the composite dose was increased further. The adsorption capacity decreases, which can be attributed to an increase in the solid–liquid ratio that directly causes this trend, since the amount adsorbed, *q_e_*, is inversely proportional to the amount of LIG/TiO_2_ [60]. In all cases, adsorption capacity was attenuated as pH increased (Figure 4c). This directly indicates a weakened electrostatic interaction of the increasingly negatively charged LIG/TiO_2_ composite with the negatively charged adsorbate. Similar trends are reported in the literature [47,61].

Thermodynamic parameters such as standard Gibbs free energy (ΔG°), enthalpy (ΔH°) and entropy (ΔS°) were used to assess the nature of the adsorption process, the spontaneity of the process, and adsorbent applicability [62]. These parameters were calculated from Equations (5) and (6), where T is the adsorption temperature and R is universal gas constant (8.314 J.mol^−1^. K^−1^). Figure 4d shows the plots of lnK_c_ against 1/T for LIG/TiO_2_ at different MO concentrations, and Table 2 lists the calculated value of each parameter.

(5) ΔG°= ΔH°− TΔS° 

(6) $$lnKc=-\frac{\Delta H{}^{\circ}\;}{RT}+\frac{\Delta S{}^{\circ}}{R}$$

**Figure 5:**
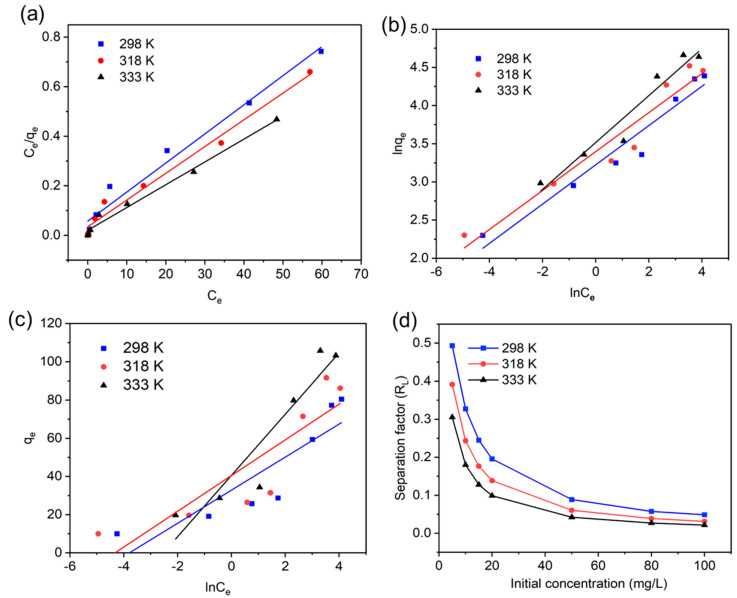
Linearized form of (**a**) a Langmuir isotherm model; (**b**) a Freundlich isotherm model; (**c**) a Temkin isotherm model; (**d**) the separation factor obtained in the adsorption experiment running at different initial concentrations of MO and temperature.

The values of the standard enthalpy (ΔH°) were in the range of 24.789–5.618 KJ/mol. Therefore, the adsorption process was an endothermic process. Physical adsorption dominates chemisorption during the adsorption of MO onto the LIG/TiO_2_, given that the initial concentration of the adsorbate is low. Additionally, positive entropy values indicate that the overall system randomness increases at the adsorbate–solution interface [63]. The negative values in ΔG° confirm that the MO adsorption onto LIG/TiO_2_ is a spontaneous process. It can also be observed that ΔG° becomes more negative at higher temperatures. This confirms that the process is endothermic, where higher temperatures favor increasing adsorption capacities. Moreover, the extent of MO uptake is temperature dependent, which agrees well with results on the adsorption of methylene blue using granular activated carbon and activated carbon fiber [64]. Figure 5a–c show the effect of temperature on the adsorption process and implies that the number of MO molecules that interact with the active sites increased with temperature [65]. This behavior can be explained by the increased kinetic energy of the MO molecules. In addition to that, MO molecules need an adequate amount of energy to influence the interaction between active sites and molecules [66].

Table 3 lists the three isotherm models used in the study, along with all the constants and coefficients of linear correlation (R^2^) values. The Langmuir model, as shown in Figure 5a, describes no transmigration of adsorbate molecules to another layer. Moreover, it assumes that the active sites have equal sorption energies [62]. The maximum adsorption capacity calculated using this model was found to be 85.1 mg.g^−1^, 92.7 mg.g^−1^, and 108.6 mg.g^−1^ at 298 K, 318 K, and 333 K, respectively. Of the three models tested, the data most closely fit the Langmuir model (R^2^ = 0.98). We thus conclude that all active sites in LIG/TiO_2_ have equal affinity to take up the MO.

Furthermore, this better fit confirms that physical sorption was the dominating mechanism. Figure 5d shows plots of the dimensionless separation factor (*R_L_*) at different initial concentrations and gave values of 0 < *R_L_*< 0.5, confirming a favorable process. Note that when *R_L_* = 0, then it is a irreversible process; when 0 < *R_L_* <1, then it is a favorable process; when *R_L_* = 1, then it is a linear process; and when *R_L_* > 1, the isotherm nature is unfavorable [67]. Equation (7) shows the relationship between the separation factor (*R_L_*) with Langmuir constant (*K_L_*) and the initial concentration of MO (*C_o_*).

(7) $$R_{L}=\frac{1}{1+K_{L}C_{o}}$$

### 3.2. Photocatalytic Degradation Efficiency

**Figure 6:**
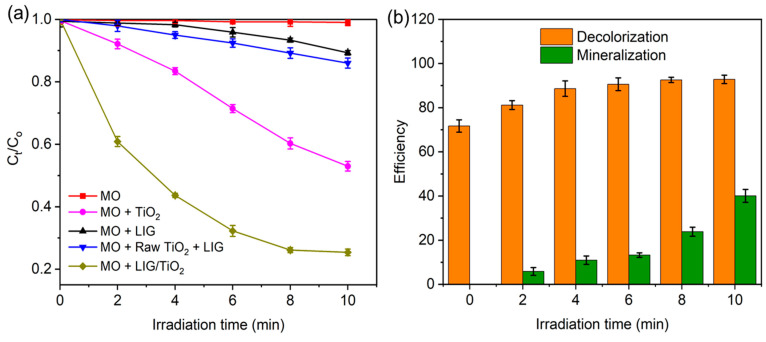
(**a**) Removal efficiency of MO using different catalysts (photocatalysis contribution only) at T = 298 K, P = 1 atm; (**b**) comparison removal efficiency and mineralization performance by LIG/TiO_2_ for an initial concentration (C_o_) of MO = 20 mg/L at standard conditions.

In light of the adsorption capability of the LIG/TiO_2_ composite catalyst, photocatalytic effects were studied. Photocatalytic oxidation reactions can use specific photon energies or a range to break down a pollutant. The rate of photocatalytic oxidation for a dye depends on its concentration, water system characteristics, and catalyst dosage. The photocatalytic activity of LIG/TiO_2_ was thus evaluated using MO, using UV light (365 nm), and was quantified by monitoring the decolorization of the dye. LIG/TiO_2_ was compared in batch mode with the following control materials: TiO_2_ only, LIG only, and the mixing of raw, unlased TiO_2_ and LIG at T = 298 K, C_o_ = 20 mg/L, catalyst dose = 500 mg/L (Figure 6a).

Under UV irradiation, MO without any catalyst showed almost no degradation (Figure 6a). In addition, LIG only showed very minor MO removal. In 10 min, UV-irradiated LIG removed only 10.8% of the initial concentration of MO. We further tested the removal efficiency of the anatase TiO_2_ and compared it to LIG alone. Using the same weight of catalyst as the weight of LIG added, we found that TiO_2_ performed 81.1% better than LIG alone. In comparison, when the catalyst was added at the same weight as in the composite LIG/TiO_2_ catalyst, the removal efficiency of the mixture of raw, unlased TiO_2_ and LIG was only slightly better than LIG alone.

On the other hand, for the LIG/TiO_2_ composite, the most MO was removed during the adsorption experiment, where the overall removal of MO in both adsorption and photocatalytic degradation was found to be 92.8% by the LIG/TiO_2_ composite. Of this 92.8%, 71.7% removal was attributed to adsorption and 21.1% because of photocatalysis effects. This drastically enhanced activity of the LIG/TiO_2_ composite compared to the catalyst mixed with LIG might be explained by LIG providing multiple conductive layers or conduits for the electrons from the embedded TiO_2_ surfaces, in which, consequently, the parasitic exciton recombination becomes lower. The formation of excitons (electrons and holes) alone does not necessarily induce photocatalytic degradation. The lower the exciton decay (electron-hole recombination) the higher the degradation performance of the material. Therefore, when merely mixed, the TiO_2_ and the LIG had a simple additive effect, whereas the drastically enhanced effect of the lased LIG/TiO_2_ composite catalyst implied a cooperative synergistic effect of the embedded TiO_2_ in LIG. This result is also in agreement with previously reported degradation studies that used a biochar-supported TiO_2_ photocatalyst for sulfamethoxazole degradation in a water system [26] and the TiO_2_/biochar-based photocatalytic degradation of methyl orange [16].

We estimated the extent of the reaction by comparing MO decolorization with total organic carbon (TOC) measurements to estimate the mineralization efficiency of the LIG/TiO_2_ composite (Figure 6b). After 2 min of photodegradation, the mineralization efficiency was 5.9%, and it increased to 40.1% after 10 min. Although many reasons might account for the high activity of the LIG/TiO_2_ composite, we further note that the LIG/TiO_2_ composite contains a mixture of anatase and rutile, in which the two forms together are known to be synergistic and more photocatalytically active than anatase alone [68]. Additionally, the band gap is slightly lower for rutile, such that the two forms in the composite would be able to absorb more light than the raw material, and this is discussed in more detail below [68]. Finally, the catalyst embedded in the graphene will enhance the effects as discussed above, and indicate a strong interaction between the LIG and TiO_2_ nanoparticles.

It has been well studied that the electrons (e^−^) in the conduction band and the holes (h^+^) in the valence band, which are generated from metal oxide semiconductors such as ZnO, produce hydroxy radicals (**•**OH) and superoxide anions (**•**O_2_^−^) upon interaction with water [20]. The literature shows H_2_O_2_ as a better electron acceptor than oxygen, and therefore H_2_O_2_ lowers oxygen recombination. Chain reactions for the mechanisms of how the semiconductor-based catalyst creates various reactive species in water system are also reported [20]. According to this principle, we devised a chain of reaction for the semiconductor under study in our research with Equations (R1)–(R7). Note, k_a_ (forward reaction) and k_d_ (backward reaction) represent adsorption and desorption constants, respectively. The values of *K_L_* given in Table 3 are the ratio of both k_a_ and k_d_ in equilibrium. The first reaction, R_1_, is a reversible reaction of adsorption and desorption. The remaining reactions (R2)–(R7) represent the photocatalysis chain reactions.

(R1) MO+LIG/TiO2⇌ kakdLIG/TiO2•MO

LIG/TiO_2_ + h*v* → h^+^ + e^−^ (R2)

h^+^ + H_2_O → H^+^ + **•**OH (R3)

e^−^ + ½ O_2_ + H^+^ → ½ H_2_O_2_ (R4)

e^−^ + O_2_ → **•**O_2_^−^ (R5)

e^−^ + H_2_O_2_ → OH^−^ + **•**OH (R6)

MO + **•**O_2_^−^/**•**OH → H_2_O + CO_2_ + other byproducts. (R7)

**Figure 7:**
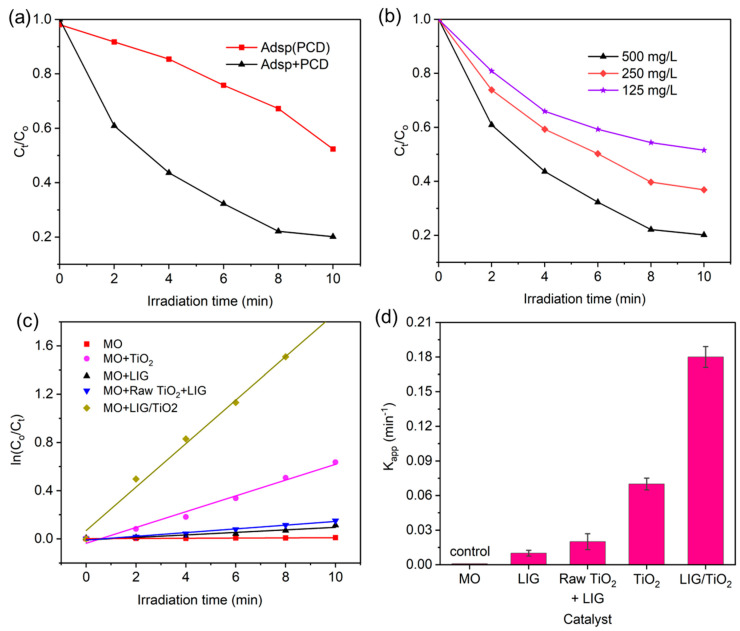
(**a**) Adsorption enhanced the photocatalytic degradation, Adsp(PCD) = adsorption and photocatalysis started both at same time (t = 0 min), Adsp + PCD = solution reaches adsorption- desorption equilibrium after 3 h, and then the photocatalysis degradation was initiated; (**b**) effect of increasing the dose of the catalyst on removal efficiency; (**c**) first order kinetics of methyl orange dye degradation for C_o_ = 20 mg/L, catalyst dose = 500 mg/L, T = 298 K, P = 1 atm.; (**d**) first order apparent kinetic constants of different catalysts.

To further investigate the adsorption enhanced photocatalytic effect, we performed an experiment where the LIG/TiO_2_ catalyst was UV irradiated immediately after mixing with MO and compared with UV irradiation only after adsorption reached equilibrium (Figure 7a). Here, the red line indicated that the adsorption and photodegradation process initiated at the same time (Adsp(PCD)) was less efficient in MO degradation compared to the black line, which indicated a sample equilibrated for 3 h protected from light, and then UV irradiated (Adsp + PCD). We conclude that the adsorption effect brings higher amounts of MO to the catalyst surface and facilitates catalytic degradation. To optimize and study the effect of the catalyst dose, degradation kinetics of MO was studied for three different doses, namely 125, 250, and 500 mg/L (Figure 7b). A higher catalyst dose led to monotonically decreased C_t_/C_o_, and overall degradation efficiency was 48.5%, 63.0%, and 80.0% for 125, 250, and 500 mg/L, respectively. Decolorization and mineralization efficiency increased with increasing LIG/TiO_2_ dosage and was attributed to the increase in available active sites.

The first order kinetic model was used to determine the rate constant of the photocatalyst (Figure 7c). The first order kinetic model is given by Equations (8) and (9) [69], where *r* is the photocatalytic reaction rate (mg/(L min)), *C* is concentration in (mg/L), *K_app_* is the apparent rate constant (min^−1^), and *t* is the irradiation time (min).

(8) $$-r=\frac{dC}{dt}=K_{app}.t$$

(9) $$\;ln\frac{Co}{Ct}=K_{app}.t$$

In Figure 7d, the apparent rate constant is shown explicitly. The value of K_app_ for each material was determined from the slope of the plot in Figure 7c. The LIG/TiO_2_ composite exhibited the highest apparent rate constant compared to other catalysts. To summarize, the K_app_ of LIG < Raw LIG + TiO_2_ < TiO_2_ < LIG/TiO_2_.

The positive impact of carbon materials can be estimated using a synergy factor (R), which is based on the apparent first order rate constant measured when carbonaceous-TiO_2_ composites and pure TiO_2_ are used, given by Equation 10 [70]. In our study, an enhanced synergy factor was seen (2.57), in comparison to similar materials such as reduced graphene oxide TiO_2_ composites, for example, which reported synergy factors between 1.35 and 1.83 [71]. It is important to note that the interpretation of relative enhancement can be tedious because it depends on the mass of TiO_2_ used (same mass in both or same overall mass). Furthermore, synergy factor results might depend on various testing techniques and tools utilized in various laboratories. Therefore, although it can give an estimate to the catalytic enhancement, one must be careful to use it as an impartial benchmark to compare findings from various studies, because the method by which the kinetic parameters are determined depends on experimental conditions such as the reaction volume, unit weight, and the area of the photocatalyst that has been exposed to radiation [31].

(10) $$R=\frac{K_{app}\left(TiO_{2}+C\right)}{K_{app}\left(TiO_{2}\right)}$$

**Figure 8:**
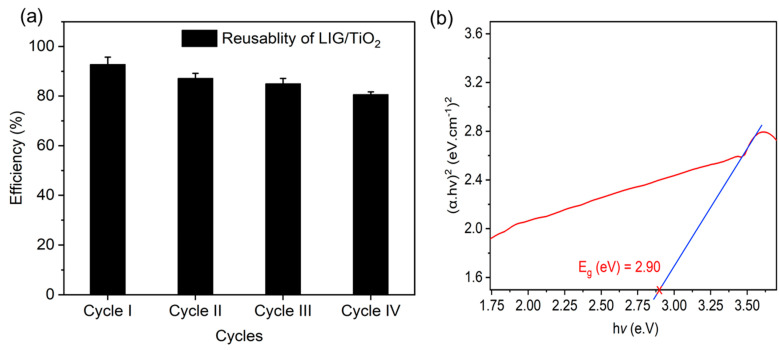
(**a**) The reusability of the LIG/TiO_2_; (**b**) band gap energy (E_g_) calculation using Tauc plot. The linear line is extrapolated to the *x*-axis and the band gap identified using an average of four fits.

Table 4 presents a comparison of selected examples of overall removal efficiency for MO. The values in the table are dependent on experimental parameters, including the concentration of dye used, the quantity of the photocatalyst, and the intensity and duration of light exposure, but give an indication that the LIG/TiO_2_ catalyst is very effective, while being fabricated using a facile and cost-effective method. Evaluating the reusability of the synthesized catalyst is also very important when evaluating the significance of a new material. Hence, the LIG/TiO_2_ composite catalyst was selected for stability and reusability performance studies. The LIG/TiO_2_ composite catalyst showed high degradation activity even after four reuse cycles (Figure 8a). Removal efficiency decreased from 92.8% in the first cycle to 80.7% in the fourth cycle. This decline in efficiency can be attributed to a decrease in adsorption sites during the successive use of the LIG/TiO_2_, and material loss due to washing.

Measuring the turnover number (TON) is also a common method used to determine the efficiency of a catalyst. However, when dealing with heterogeneous catalysts the TON is normalized over time, resulting in the turnover frequency (TOF; mmol mg^–1^ s^–1^) as the calculated output [34].

(11) $$TOF=\frac{N_{MO}}{t\;.M_{LIG/TiO_{2}}}$$

The amount of feed components is shown above by “*N_MO_*” in mmol, whereas the *LIG*/*TiO*_2_ composite is denoted by the letter “*M*” and “*t*” represents the irradiation time. In this study, the estimated *TOF* was reported to be 2.02 × 10^−7^ mmol mg^−1^ s^−1^.

Figure 8b presents the estimated band gap of the composite. In semiconductors, the band gap energy describes the amount of energy required to excite an electron from its valence band to conduction band. Determining the accurate band gap of the semiconductor is important for estimating the photochemical and photophysical properties of the material [77,78,79,80]. TiO_2_ has a relatively large band gap: ~3.2 eV for anatase phase and ~3.0 eV for its rutile phase [80]. The Tauc method was used to calculate the band gap of our composite. According to Tauc, the energy dependent absorption coefficient α is given in the following Equation (12), where *v* is photon′s frequency, *h* is Plank’s constant, *Eg* is band gap energy and *B* is a constant. The value of γ is dependent on the nature of electron transitions, and is ½ or 2 for direct and indirect band gap transitions, respectively [78]. As shown in Figure 8b, we have calculated the band gap of LIG/TiO_2_ to be 2.90 ± 0.06 eV.

(α. hv)^1/γ^ = B(hv—Eg) (12)

This indicates that the material can be used at the short wavelength visible region, and underlines that the lasing and embedding of the TiO_2_ catalyst in LIG can result in a lowering of the band gap and is an important factor in the increased efficiency of the photocatalyst.

## 4. Conclusions

In this study, we demonstrated the preparation and characterization of a new LIG/TiO_2_ composite photocatalyst for the removal of organic pollutants. The lasing procedure resulted in an LIG composite catalyst with mixed rutile and anatase forms of embedded TiO_2_, a reduced band gap, and adsorption enhanced photocatalytic activity. Overall, the LIG/TiO_2_ composite exhibited excellent removal and mineralization of MO in different operating conditions and can be reused. As MO can be a model compound for organic pollutants, the LIG/TiO_2_ composite could be extended in the future for other contaminants’ removal. Adsorption enhanced photodegradation could lead to the targeted degradation of pollutants, given that various organic or inorganic materials would have variable binding affinities to the LIG. Testing in real water conditions or tuning of the binding affinity toward specific emerging contaminants are, therefore, concepts that might warrant further study. Furthermore, the fabrication method used here can be used for the synthesis of other carbonaceous-metal oxide composites that might be used for a wide range of applications in the fields of catalysis, battery, and energy conversion.

## Figures and Tables

**Table 1:** List of the adsorption isotherm models used and their expressions.

Isotherm Model	Equation	Plotting
Langmuir	$\frac{1}{q_{e}}=\frac{1}{q_{max}\;K_{L}}\frac{1}{C_{e}}+\frac{1}{q_{max}}$	*1/q_e_* vs. *1/C_e_*
Freundlich	$lnq_{e}=\frac{1}{n}ln\;C_{e}+ln\;K_{f}$	*ln q_e_* vs. *ln C_e_*
Temkin	*q_e_ =* $\;\frac{RT}{b}$ *ln C_e_ +* $\frac{RT}{b}$ *ln A_T_*	*q_e_* vs. *ln C_e_*

**Table 2:** Thermodynamic parameters of MO adsorption onto LIG/TiO_2_.

Adsorbate	Concentration	ΔS°	ΔH°		−ΔG°		
	(mg/L)	(KJ/mol.K)	(KJ/mol)		(KJ/mol)		
				298 K	318 K	333 K	R^2^
	5	59.931	24.789	4.382	4.997	5.930	0.865
MO	20	19.932	8.122	0.176	0.703	1.620	0.964
	50	17.318	7.431	2.401	0.228	0.883	0.998
	80	17.067	5.618	6.700	3.236	1.120	0.985

**Table 3:** Calculated values of adsorption isotherm parameters at different temperatures.

Isotherm Model	Parameters	Temperature (K)		
		298	318	333
Langmuir	q_max_ (mg.g^−1^)	85.110	92.680	108.58
	*K_L_* (L.mg^−1^)	0.210	0.311	0.455
	*R_L_*	0.195	0.138	0.090
	R^2^	0.981	0.981	0.987
Freundlich	K_f_	25.11	29.87	33.64
	1/n	0.26	0.25	0.31
	n	3.88	3.92	3.26
	R^2^	0.944	0.931	0.943
Temkin	A_T_	44.23	75.45	12.53
	B (J.mol^−1^)	8.66	9.35	16.03
	b	286.2	264.9	154.6
	R^2^	0.783	0.767	0.886

**Table 4:** Comparison of overall MO removal efficiency of different materials.

Irradiation	Photocatalyst	Overall Removal Efficiency (%)	Reference
UV-A light	TC0 ^a^, TC62.5 ^b^	45.5,62.9	[72]
Visible light	Sn-ZnO/GO	87	[73]
Visible light	ZnO-TiO_2_/SO_4_^2−^	90	[74]
Visible light	CoOx/g-C_3_N	92	[75]
Visible light	SbSI MRs	78	[76]
UV-A light	LIG/TiO_2_	92.8	Present study

^a^ Titanium dioxide without carbon dots; ^b^ titanium dioxide with high loading of carbon dots.

## Supplementary Materials

The following supporting information can be downloaded at: https://www.mdpi.com/article/10.3390/nano13050947/s1, Figure S1: Images of PI substrate, laser parameters, and laser system; Figure S2: Images of LIG/TiO_2_ fabrication process; Figure S3: TEM images of LIG/TiO_2_ composite; Figure S4: XRD of LIG/TiO_2_ composite; Figure S5: Adsorption kinetic models.
