# Supplementary material for: A Laser-Induced Graphene-Titanium(IV) Oxide Composite for Adsorption Enhanced Photodegradation of Methyl Orange
Source: Nanomaterials (Basel). 2023 Mar 5;13(5):947. doi: 10.3390/nano13050947 (PMC10005721; doi:10.3390/nano13050947)
Supplement: Supplementary file 1 [file nanomaterials-13-00947-s001.zip › nanomaterials-2235233-supplementary.pdf]

Supplementary Information

A Laser-Induced Graphene-Titanium (IV) Oxide Composite for Adsorption Enhanced Photodegradation of Methyl Orange

Brhane A. Tesfahunegn, Maurício Nunes Kleinberg, Camilah D. Powell and Christopher J. Arnusch *

Department of Desalination and Water Treatment, Zuckerberg Institute for Water Research,

The Jacob Blaustein Institutes for Desert Research, Ben-Gurion University of the Negev,

Midreshet Ben-Gurion 8499000, Israel

* Corresponding Author Details:

Dr. Christopher J. Arnusch

Department of Desalination and Water Treatment

Zuckerberg Institute for Water Research

The Jacob Blaustein Institutes for Desert Research

Ben-Gurion University of the Negev

Sde Boqer Campus

8499000 Midreshet Ben-Gurion, Israel

Phone: +972-8-656-3532

Email: arnusch@bgu.ac.il

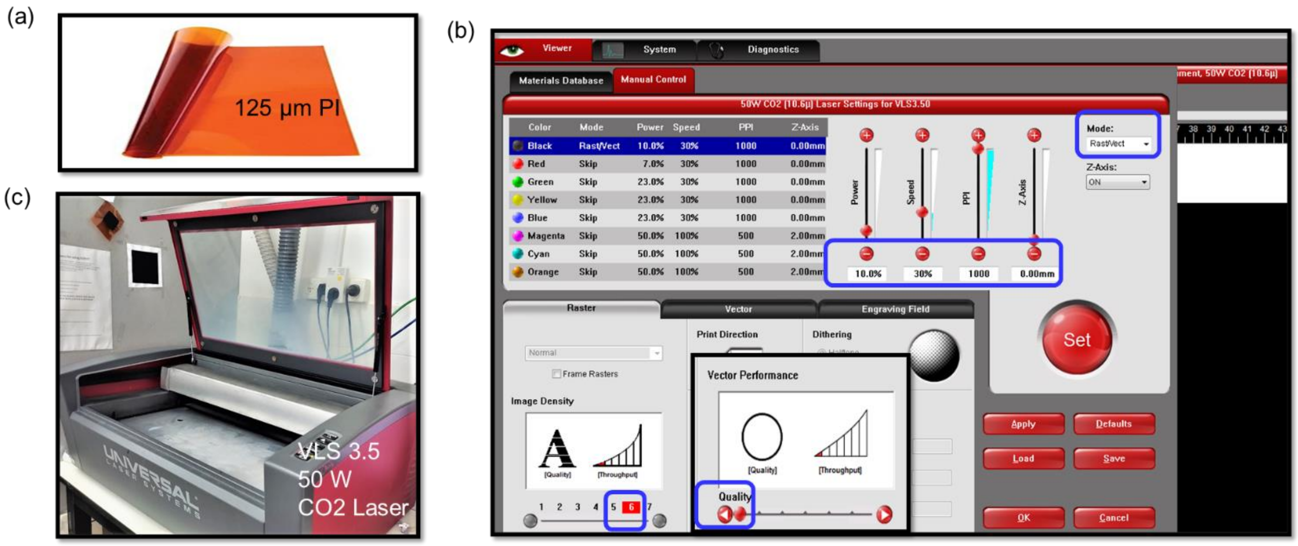

Figure S1. (a) PI substrate; (b) Laser parameters used during LIG/TiO_2 fabrication as viewed in the software of the VLS 3.50 laser from Universal Laser Systems; (c) Photograph of the laser system used.

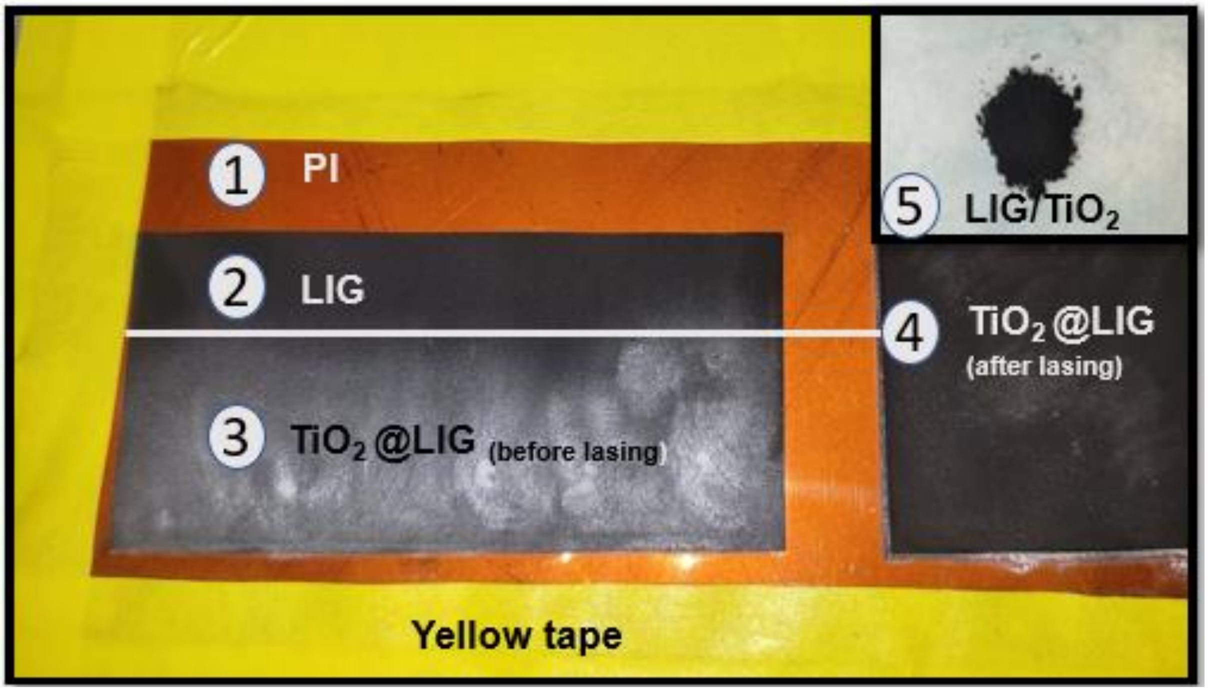

Figure S2. LIG/TiO_2 fabrication process, showing (1) the polyimide (PI) substrate, (2) LIG fabricated on the PI substrate, (3) TiO_2 coated LIG (before second lasing), (4) LIG/TiO_2 composite after second lasing, and (5) the LIG/TiO_2 powder obtained after scraping the composite from the PI surface.

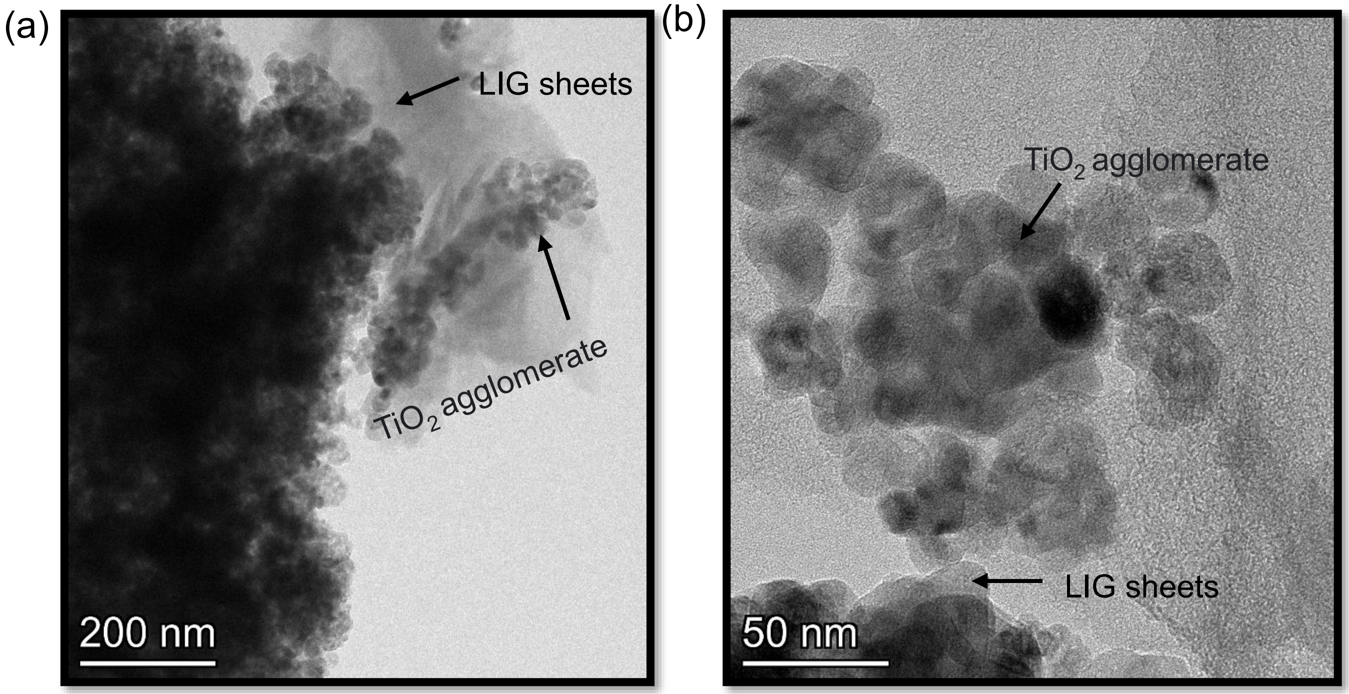

Figure S3. TEM images of LIG/TiO_2 composite. (a) TEM image showing both LIG sheets and TiO_2 nanoparticles and agglomerates, scale bar 200 nm; (b) TEM image at a higher magnification showing both LIG sheets and TiO_2 nanoparticles and agglomerates scale bar 50 nm.

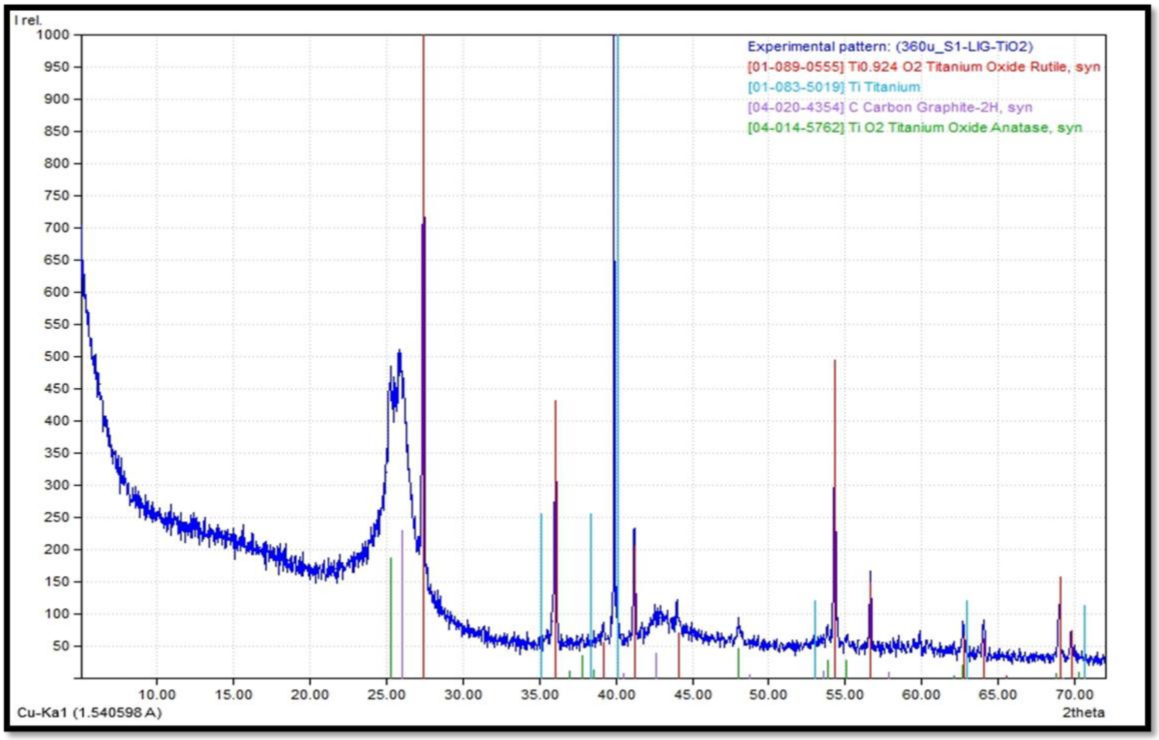

Figure S4. XRD phase/composition identification of LIG/TiO_2 sample.

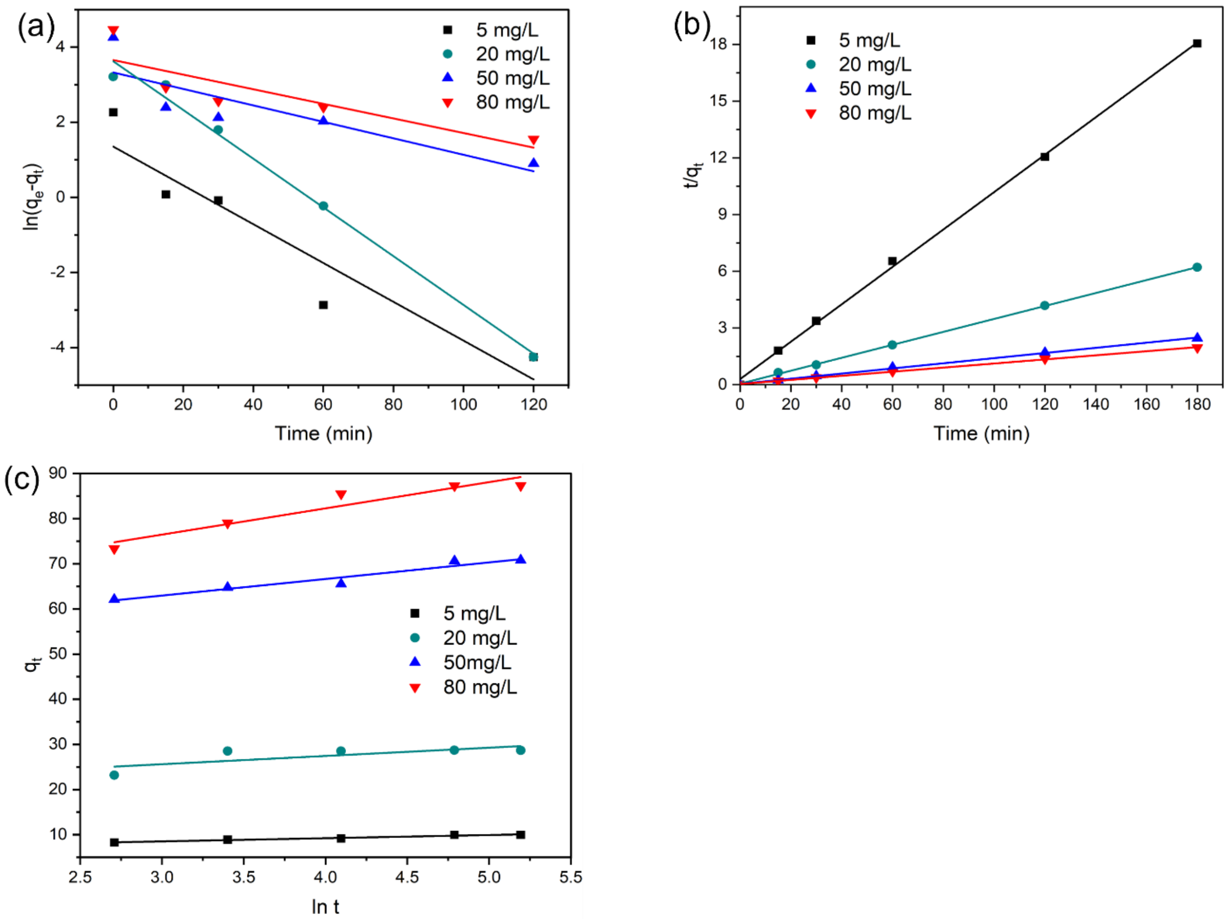

Figure S5. Adsorption kinetic models (a) Pseudo first order; (b) Pseudo second order; (c) Elovich
